# Dynamics of the Microbial Community and Opportunistic Pathogens after Water Stagnation in the Premise Plumbing of a Building

**DOI:** 10.1264/jsme2.ME21065

**Published:** 2022-03-24

**Authors:** Iftita Rahmatika, Futoshi Kurisu, Hiroaki Furumai, Ikuro Kasuga

**Affiliations:** 1 Department of Urban Engineering, Graduate School of Engineering, The University of Tokyo, Tokyo, Japan; 2 Research Center for Water Environment Technology, Graduate School of Engineering, The University of Tokyo, Tokyo, Japan

**Keywords:** drinking water, microbial regrowth, opportunistic pathogens, premise plumbing, water stagnation

## Abstract

In premise plumbing, microbial water quality may deteriorate under certain conditions, such as stagnation. Stagnation results in a loss of disinfectant residual, which may lead to the regrowth of microorganisms, including opportunistic pathogens. In the present study, microbial regrowth was investigated at eight faucets in a building over four seasons in one year. Water samples were obtained before and after 24 h of stagnation. In the first 100‍ ‍mL after stagnation, total cell counts measured by flow cytometry increased 14- to 220-fold with a simultaneous decrease in free chlorine from 0.17–0.36‍ ‍mg L^–1^ to <0.02‍ ‍mg L^–1^. After stagnation, total cell counts were not significantly different among seasons; however, the composition of the microbial community varied seasonally. The relative abundance of *Pseudomonas* spp. was dominant in winter, whereas *Sphingomonas* spp. were dominant in most faucets after stagnation in other seasons. Opportunistic pathogens, such as *Legionella pneumophila*, *Mycobacterium avium*, *Pseudomonas aeruginosa*, and *Acanthamoeba* spp., were below the quantification limit for real-time quantitative PCR in all samples. However, sequences related to other opportunistic pathogens, including *L. feeleii*, *L. maceachernii*, *L. micdadei*, *M. paragordonae*, *M. gordonae*, and *M. haemophilum*, were detected. These results indicate that health risks may increase after stagnation due to the regrowth of opportunistic pathogens.

Biological stability is a critical aspect in drinking water supply systems (Prest *et al.*, 2016). To achieve biological stability and prevent the growth of microorganisms, including pathogens, a disinfectant residual is generally maintained throughout the drinking water distribution system in several countries, including Japan. In Japan, free chlorine (>0.1‍ ‍mg L^–1^) or combined chlorine (>0.4‍ ‍mg L^–1^) must be maintained at the end of the pipe. However, stagnation, which is defined as intermittent water use at individual outlets for an extended period, may occur in premise plumbing, causing longer incubation times (Proctor and Hammes, 2015; Rhoads and Hammes, 2021). It reduces disinfectant levels, allows microbial regrowth, and, thus, changes the microbial community composition in drinking water (Lautenschlager *et al.*, 2010; Zhang *et al.*, 2021b).

Premise plumbing is characterized by a small-diameter pipe with a high surface area to volume ratio, in which disinfection decay may be faster than in the water main (Hoekstra and Tsvetanova, 2010; Ling *et al.*, 2018). Some pipe materials release biodegradable organic matter, which further promotes the growth of planktonic bacteria and the development of biofilms on pipe surfaces (Wen *et al.*, 2015; Rahmatika *et al.*, 2020). Previous studies demonstrated that stagnation contributed to bacterial proliferation. Intact cell counts and heterotrophic plate counts (HPC) were shown to increase after overnight stagnation in premise plumbing (Lautenschlager *et al.*, 2010). Furthermore, the significant decay of free chlorine after stagnation caused total cell counts (TCC) to increase (Ling *et al.*, 2018; Zhang *et al.*, 2021b). Increases in water temperature and biofilm detachment were also found to promote microbial growth during stagnation (Zlatanovic *et al.*, 2017; Bedard *et al.*, 2018; Peng *et al.*, 2020; Zhang *et al.*, 2021a).

Previous studies characterized microbial community compositions in premise plumbing and evaluated their dynamics under stagnation (Lautenschlager *et al.*, 2010; Ling *et al.*, 2018; Zhang *et al.*, 2021b). Some genera, including *Methylobacterium* spp., *Sphingomonas* spp., *Sphingobium* spp., *Mycobacterium* spp., and *Pseudomonas* spp., increased after stagnation (Zhang *et al.*, 2021a; 2021b). Ley *et al.* (2020) reported a positive correlation between *Legionella* spp. and stagnation times (Ley *et al.*, 2020). Multiple studies have linked the occurrence of premise plumbing-associated opportunistic pathogens (*i.e*., *L. pneumophila*, *M. avium*, and *P. aeruginosa*) to water-borne infections, including Legionnaires’ disease, Pontiac fever, and nontuberculous pulmonary disease (Anaissie *et al.*, 2002; Falkinham *et al.*, 2008; Wang *et al.*, 2013).

Regardless of the health risks of decreased water quality associated with microbial regrowth in premise plumbing, microbial water quality after stagnation is not routinely monitored. Moreover, limited information is currently available on the factors contributing to variations in the microbial water quality of different faucets in the same building. A more detailed understanding of the characteristics of the drinking water microbiome after stagnation is crucial for developing effective strategies that ensure the safety of water for consumption. In the present study, one-year monitoring at different faucets in a building was performed to reveal the dynamics of the microbial community before and after stagnation. In addition, the occurrence of opportunistic pathogens was analyzed to assess potential health risks.

## Materials and Methods

### Study sites

The present study was conducted at an 11-story building at the University of Tokyo, Tokyo, Japan. Drinking water samples were collected from eight cold water faucets (F1–F8) at the handwashing basins in laboratory rooms. The target faucets were located on the 3^rd^ and 4^th^ floors. The premise plumbing in that building was constructed using steel with a polyethylene powder lining. It has been used since 1995 when the building was constructed. The building stores drinking water in rooftop tanks (8‍ ‍m^3^), which is distributed to the faucets on each floor. Water is supplied from a water treatment plant that treats surface water by coagulation followed by sedimentation, primary rapid sand filtration, ozonation, biological activated carbon filtration, and secondary rapid sand filtration. The typical free chlorine concentration in treated water at the plant is 0.5–0.7‍ ‍mg L^–1^ to maintain a free chlorine residual at faucets of >0.1‍ ‍mg L^–1^, which is regulated by the Japanese drinking water quality guidelines.

### Sample collection

To evaluate seasonal variations in microbial regrowth, pre- and post-stagnation samples were collected in four seasons. Samples were collected from eight faucets (F1–F8) in summer (June–July 2018) and from six faucets (F1, F3, F4, F5, F7, and F8) in autumn (November 2018), winter (January 2019), and spring (April 2019) (52 samples) (Table S1). Prior to designated stagnation, a 10-L pre-stagnation sample was collected after flushing water for 5‍ ‍min at an approximate flow rate of 5 L‍ ‍min^–1^. The pre-stagnation sample represents drinking water samples with the minimized influence of stagnation. Afterwards, the faucets were closed for 24 h. Post-stagnation samples were collected from the same faucet after approximately 24 h of stagnation at a flow rate of approximately 5 L‍ ‍min^–1^. The first 2 L of the flushed post-stagnation sample was collected in incremental samples of 100‍ ‍mL.

Daily variations in stagnation-induced microbial growth were evaluated by repeating the above sample collection procedure at F1 on six consecutive days in April 2019.

### Water quality ana­lysis

Temperature and free chlorine were measured with a digital thermometer (SK-250WP II-R; Sato Keiryoki) and HACH chlorine pocket colorimetric according to the *N,N*-diethyl-*p*-phenylenediamine method (Hach), respectively. The limit of quantification (LOQ) of the free chlorine measurement was 0.02‍ ‍mg L^–1^.

### Bacterial abundance

TCC were analyzed by flow cytometry. SYBR^®^ Green I (Invitrogen) diluted 100-fold with dimethylsulfoxide (FUJIFILM Wako Pure Chemical) was used as a working dye solution. Then, 490‍ ‍μL of the sample was stained with 5‍ ‍μL of the working dye solution and 5‍ ‍μL of 0.5 mol L^–1^ EDTA (Invitrogen). In the ana­lysis, 0.2-μm filtered samples were used as a blank. This mixture was vortexed and incubated at 37°C for 10‍ ‍min under dark conditions and analyzed using a BD Accuri C6^®^ flow cytometer (BD Biosciences). Electronic gating for bacterial cell quantification was selected on density plots of green fluorescence in the FL1 channel (535±15‍ ‍nm) and red fluorescence in the FL3 channel (>670‍ ‍nm) (Fig. S1). Constant gating was applied for all samples.

HPC were enumerated using the spread plate method following Standard Method 9215 C (APHA, 1998). Water samples were serially diluted in autoclaved phosphate-buffered saline (0.01 mol L^–1^, pH 7.2–7.4) (FUJIFILM Wako Pure Chemical). One hundred-microliter aliquots of non-diluted or diluted samples were spread on R2A agar medium (Beckton, Dickinson) and incubated at 20°C for 7 days.

### DNA extraction

To collect the biomass from samples, the first 1 L of post-stagnation samples was filtered through 0.2-μm polycarbonate filters (Isopore; Merck Millipore). Regarding pre-stagnation samples, 10 L of samples were concentrated using a hollow fiber ultrafiltration (HFUF) unit with a surface area of 25 m^2^ (APS-25SA; Asahi Kasei Medical), following a previously described method (Liu *et al.*, 2020). The recovery ratio of *E. coli* cells by HFUF was reported as 96.5±8.5% (Liu *et al.*, 2020). Prior to the filtration process using a HFUF unit, a blocking process was conducted by circulating 5% fetal bovine serum (Thermo Fisher Scientific) in the HFUF cartridge for 2‍ ‍min. The HFUF cartridge containing fetal bovine serum was incubated at room temperature overnight. The sample volume was concentrated from 10 L to approximately 150‍ ‍mL by applying the dead-end filtration method using a rotary pump at a flow rate of 1.0 L‍ ‍min^–1^. After filtration, the concentrates in the filter were back-flushed into a sterile bottle and mixed with 100× elution buffer (10% Tween 80, 1% sodium polyphosphate, and 0.1% antifoam A) (Sigma-Aldrich). The mixture of concentrates and elution buffer was circulated for 2‍ ‍min to further recover cells from the HFUF cartridge. The final concentrated sample was filtered through a 0.2-μm polycarbonate membrane filter. Filters were stored at –20°C until DNA extraction.

DNA was extracted from the filter using the FastDNA SPIN Kit for Soil (MP Biomedicals) following the manufacturer’s instructions. Briefly, the filter was placed in a lysing matrix tube, and dissolved in phenol–chloroform–isoamyl alcohol (25:24:1) (Nippon Gene). Bead beating was performed with Fast-Prep (MP Biomedicals) at a speed of 6.0‍ ‍m s^–1^ for 40 s. The DNA extraction efficiency of this method evaluated using *E. coli* was 93.8±4.9% (*n*=3). A negative control experiment using milli-Q water revealed that DNA contamination was negligible.

### Real-time quantitative PCR (qPCR)

The number of gene copies of *Legionella* spp. (23S-5S rRNA spacer region), *L. pneumophila* (*mip* gene), *Mycobacterium* spp. (16S rRNA gene), *M. avium* (16S rRNA gene), *Pseudomonas aeruginosa* (*regA* gene), and *Acanthamoeba* spp. (18S rRNA gene) were quantified using TaqMan-based qPCR (Herpers *et al.*, 2003; Riviere *et al.*, 2006; Feazel *et al.*, 2009; Gensberger *et al.*, 2013). The primers and probes used for target genes and thermal conditions are described in Table S2. Each reaction mixture (20‍ ‍μL) consisted of 7.7‍ ‍μL of distilled water, 10‍ ‍μL of the LightCycler^®^ Probes Master solution (Roche Life Science), 0.1‍ ‍μL of each primer (100 pmol μL^–1^), 0.1‍ ‍μL of the probe (40 pmol μL^–1^), and 2‍ ‍μL of the DNA template. Standards were prepared using a 10-fold serial dilution (5.0×10^0^–5.0×10^6^ copies μL^–1^) of an artificially synthesized plasmid containing the target genes (Table S3). LOQ was set as the lowest concentration of the standards. All reactions were performed in triplicate using a LightCycler^®^ LC480 system (Roche Life Science). PCR reaction efficiencies were in the range of 90.15–99.50% (Table S2). PCR inhibition was not observed.

### Amplicon sequencing of 16S rRNA genes

The microbial community structure was analyzed using amplicon sequencing targeting the V4 region of 16S rRNA genes. The V4 regions of bacterial and archaeal 16S rRNA genes were amplified by primers with adapter sequences (underlined): 515F (5′-ACACTCTTTCCCTACACGACGCTCTTCCGATCT-GTGCCAGCMGCCGCGGTAA-3′) and 806R (5′-GTGACTGGAGTTCAGACGTGTGCTCTTCCGATCT-GGACTACHVGGGTWTCTAAT-3′) (Caporaso *et al.*, 2011). The thermal condition of the first PCR consisted of 94°C for 2‍ ‍min, followed by 25 cycles of 94°C for 30‍ ‍s, 50°C for 30‍ ‍s, and 72°C for 30‍ ‍s, with a final extension at 72°C for 5‍ ‍min. TaKaRa Ex-Taq HS (TaKaRa Bio) was used for the PCR reaction. The second PCR and amplicon sequencing based on an Illumina MiSeq platform with 300-bp paired-end reads were performed at the Bioengineering Lab, Japan. FASTX-Toolkit (ver. 0.0.14) (http://hannonlab.cshl.edu/fastx_toolkit/) was applied to filter reads whose sequences had a perfect match with the primer sequences. The deletion of primer sequences, paired-end reads under 250 bases, and low qualified bases and the merging of paired-end reads were processed using Quantitative Insights into Microbial Ecology 2 (QIIME 2) (Bolyen *et al.*, 2019) with parameters in default conditions. DADA2 in the QIIME 2 pipeline (ver. 2019.10) was used to remove chimeric and noisy sequences. Representative sequences were clustered into amplicon sequence variants (ASVs) with 100% similarity by QIIME 2. Final ASVs were taxonomically classified by referring to the EzBioCloud 16S database (May 2018) (Yoon *et al.*, 2017).

In addition to the Illumina MiSeq platform, DNA from 12 post-stagnation samples, which showed that the relative abundance of *Legionella* or *Mycobacterium* was >1% of the total microbial community, were analyzed using nanopore sequencing targeting full-length 16S rRNA genes. The library for the 16S rRNA gene ana­lysis was prepared using the 16S Barcoding Kit (Oxford Nanopore Technologies). Primers with adapter sequences (underlined) were used to amplify full-length 16S rRNA genes: 16S-F (5′-ATCGCCTACCGTGAC-Barcode-AGAGTTTGATCMTGGCTCAG-3′) and 16S-R (5′-ATCGCCTACCGTGAC-Barcode-CGGTTACCTTGTTACGACTT-3′). The thermal conditions of the first PCR consisted of 94°C for 2‍ ‍min, followed by 30 cycles of 94°C for 30‍ ‍s, 55°C for 30‍ ‍s, and 65°C for 80‍ ‍s, with a final extension at 65°C for 5‍ ‍min. LongAmp Taq 2× Master Mix (New England BioLabs) was used for the PCR reaction. The library was loaded onto R9.4 flow cells on the GridION platform (Oxford Nanopore Technologies) at the Bioengineering Lab, Japan. Base-calling and de-barcoding were performed using GUPPY software (ver. 3.2.6) (Oxford Nanopore Technologies). Adapter sequences were trimmed using Porechop (ver. 0.2.3). The SeqIO module in Biopython was used to extract reads with 1,400–1,600 bp. After subsampling by Filtlong software (ver 0.2.0), 221,978–678,877 sequences per sample were obtained. The taxonomic assignment was performed with RDP_16S_V16_sp using Usearch (ver. 11.0.667).

All sequence data in the present study were submitted to the DNA Data Bank Japan (DDBJ) Sequence Read Archive and are available under accession number DRA011523.

### Statistical ana­lysis

Statistical ana­lyses of TCC, HPC, and qPCR data were performed after log transformation. The paired Student’s *t*-test and an ana­lysis of variance (ANOVA) followed by Tukey’s honestly significant difference test (HSD) were performed using R statistical software (ver. 3.4.1), and *P*<0.05 was considered to be significant. The relevant statistical test results, including *P* values, F values, and 95% CI, are shown in Table S5, S6, and S7.

Alpha diversity indices (observed richness and the Shannon diversity index) were calculated with the vegan package (ver. 2.5-7) in R statistical software. To evaluate the significance of differences in alpha-diversity indices between sample groups, the paired Student’s *t*-test, an ANOVA, and HSD test were performed. To identify any significant differences (*P*<0.05) in the microbial community between sample groups, an ana­lysis of similarity (ANOSIM) was performed based on the Bray–Curtis dissimilarity index at the OTU level using the vegan package in R statistical software with 999 permutations. The value generated from random permutations (R value) ranged between 0 (complete similarity) and 1 (complete dissimilarity). Additionally, hierarchical clustering at the OTU level was created with the vegan package (ver. 2.5-7) in R statistical software based on the Bray–Curtis dissimilarity index.

## Results

### Example of a microbial regrowth event observed at a faucet

**Fig. 1.**
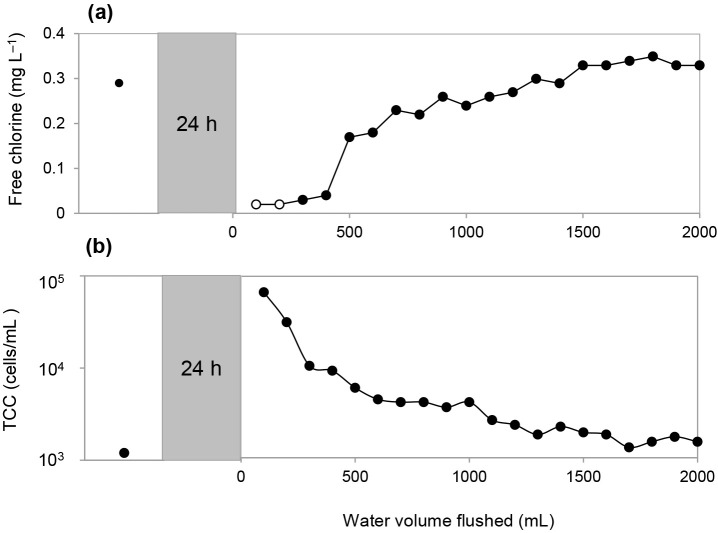
Changes in the (a) free chlorine concentration and (b) total cell counts before and after 24 h of stagnation on day 1 of the stagnation experiment at F1. Open circle symbols indicate the limit of quantification of free chlorine (0.02‍ ‍mg L^–1^).

Fig. 1 shows an example of microbial regrowth observed at F1. The pre-stagnation sample contained 0.29‍ ‍mg L^–1^ free chlorine and 2.1×10^3^‍ ‍cells‍ ‍mL^–1^ TCC. After 24 h of stagnation, free chlorine in the first 100‍ ‍mL decreased to <0.02‍ ‍mg L^–1^, while TCC increased to 5.5×10^4^‍ ‍cells‍ ‍mL^–1^. The temperature pre-stagnation was 18.3°C and increased to 20.7°C in the first 100‍ ‍mL after 24 h of stagnation (Table S4). TCC gradually decreased to 1.2×10^4^‍ ‍cells‍ ‍mL^–1^ after flushing 500‍ ‍mL water, and reached 4.0×10^3^‍ ‍cells‍ ‍mL^–1^ after flushing 2 L. Simultaneously, the free chlorine concentration recovered to 0.17‍ ‍mg L^–1^ after flushing 500‍ ‍mL water and reached 0.33‍ ‍mg L^–1^ after flushing 2 L. Daily variations in microbial regrowth after 24 h of stagnation were assessed on six consecutive days at F1. TCC levels after stagnation were 5.2±0.5×10^4^‍ ‍cells‍ ‍mL^–1^ (average±standard deviation, *n*=6) (Fig. S2a). Microbial community structures after repetitive stagnation were similar (Fig. S2b). These results indicated that the regrowth event at the same faucet was reproducible in the short term.

### Seasonal variations in microbial regrowth

**Fig. 2.**
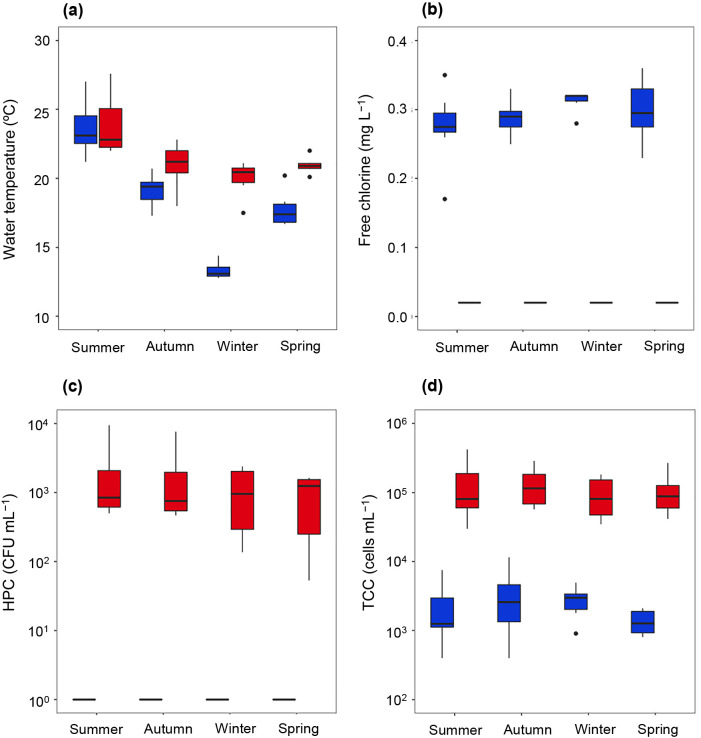
Seasonal variations in (a) water temperature, (b) free chlorine, (c) heterotrophic plate counts, and (d) total cell counts in (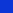) pre- and (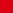) post-stagnation samples (first 100‍ ‍mL) collected in four seasons.

Fig. 2 summarizes variations in water temperature, free chlorine, TCC, and HPC in all samples collected during different seasons. The increase in water temperature in the first 100‍ ‍mL of samples collected after stagnation was observed for most samples (Fig. 2a). Differences in water temperature before and after stagnation were significant (paired Student’s *t*-test, *P*=3.059×10^–5^), with the highest increase being 8.3°C for an F1 sample collected during winter (Table S4). While the free chlorine of pre-stagnation samples (0.17–0.36‍ ‍mg L^–1^) was not significantly different among seasons, as confirmed with ANOVA test (*P*=0.365), the significant depletion of free chlorine to <0.02‍ ‍mg L^–1^ in the first 100‍ ‍mL sampled after stagnation (paired Student’s *t*-test, *P*=9.738×10^–23^) was consistently observed after stagnation for all samples (Fig. 2b).

Culturable bacteria were not detected in pre-stagnation samples (Fig. 2c). HPC increased in the first 100‍ ‍mL collected after stagnation across all samples. HPC in post-stagnation samples were 1.7±2.2×10^3^ CFU mL^–1^, and differences among seasons were not significant (ANOVA, *P*=0.540). Several samples, such as F1 (summer, autumn, and winter) and F3 (summer, autumn, and winter), exceeded the target levels for drinking water quality in Japan (2,000 CFU mL^–1^) (Table S4). TCC in pre-stagnation samples were 2.7±2.5×10^3^‍ ‍cells‍ ‍mL^–1^ and did not significantly different among seasons (ANOVA, *P*=0.521) (Fig. 2d). A significant increase in TCC from 2.7±2.5×10^3^ to 1.2±0.9×10^5^‍ ‍cells‍ ‍mL^–1^ in the first 100‍ ‍mL of water collected after stagnation was observed for all samples collected in all four seasons (paired Student’s *t*-test, *P*=8.598×10^–21^). Average TCC levels post-stagnation were the highest in summer; however, differences were not significant (ANOVA, *P*=0.736). TCC levels peaked in the first 100‍ ‍mL of water collected after stagnation in all samples. The first 100‍ ‍mL contributed to 10–47% of all cells discharged in the first 2 L of post-stagnation samples.

Microbial community structures in pre- and post-stagnation samples were analyzed. An amplicon sequencing ana­lysis revealed that 1,591 and 872 OTUs were recovered from pre- and post-stagnation samples, respectively, ranging between 110 and 339 OTUs per sample. Alpha diversity measured by OTU richness and the Shannon diversity index showed seasonal trends in pre- and post-stagnation samples (Fig. S3). Winter samples exhibited significantly lower OTU richness in pre-stagnation samples than autumn samples, as confirmed by Tukey’s HSD test (ANOVA, *P*=0.0299; Tukey’s HSD, *P*=0.039). OTU richness in post-stagnation samples in winter was significantly lower than in summer and spring (ANOVA, *P*=8.300×10^–4^; Tukey’s HSD, *P*=4.334×10^–4^–0.045). In winter, microbial communities pre- and post-stagnation were also less diverse; the Shannon index was significantly lower in winter than in other seasons (ANOVA, *P*=0.014 and 0.015, respectively; Tukey’s HSD, *P*=0.001–0.053). Average OTU richness decreased after stagnation in all seasons; however, the decrease was not significant in spring (paired Student’s *t*-test, *P*=0.303).

**Fig. 3.**
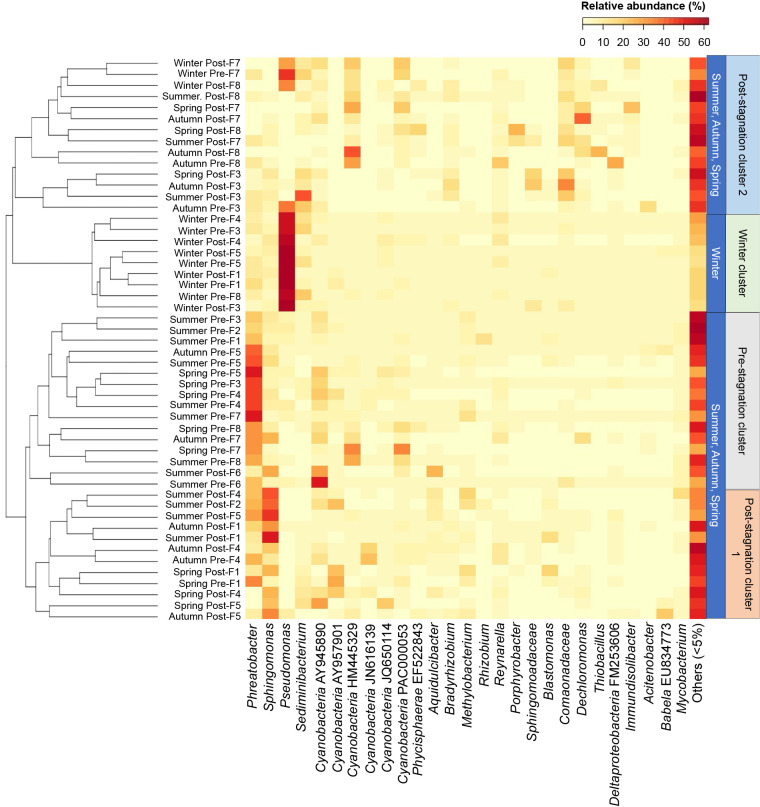
Cluster ana­lysis and heatmap of the microbial community at the genus level in pre- and post-stagnation samples in four different seasons. Pre- and post-indicate pre-and post-stagnation (first 1 L) samples, respectively.

To compare the microbial community composition pre- and post-stagnation among different seasons, a cluster ana­lysis based on the Bray–Curtis dissimilarity index was conducted (Fig. 3). Four major clusters were obtained as follows: (i) pre-stagnation cluster, (ii) post-stagnation cluster 1 (the majority of post-stagnation samples from F1, F2, F4, F5, and F6), (iii) post-stagnation cluster 2 (the majority of post-stagnation samples from F3, F7, and F8), and (iv) winter cluster. A clustering ana­lysis highlighted differences in microbial community structures pre- and post-stagnation in winter. A pairwise test using ANOSIM also supported significant differences in microbial communities pre- and post-stagnation between winter and other seasons (R value=0.3981–1.0000, *P*<0.05) (Table S8).

The pre-stagnation cluster consisted of the most pre-stagnation samples in summer, autumn, and spring, in which *Phreatobacter* spp. were predominant with a relative abundance of 15–46%. The abundance of this genus was significantly lower in winter (1–3%). The shift in the microbial community after stagnation in F1, F2, F4, F5, and F6 in summer, autumn, and spring was characterized by a predominance of *Sphingomonas* spp. The microbial community after stagnation from these samples was clustered in post-stagnation cluster 1. *Phreatobacter* spp. decreased in abundance from 27 to 8%, while *Sphingomonas* spp. increased from 5 to 22%. On the other hand, the shift in dominant bacteria in F3, F7, and F8 after stagnation in summer, autumn, and spring (post-stagnation cluster 2) was generally characterized by genera in *Comamonadaceae*, *Dechloromonas* spp., *Cyanobacteria*, and *Porphyrobacter* spp. Differences in the microbial communities of post-stagnation samples between these two groups of samples in these seasons were supported by ANOSIM (R value=0.9049, *P*<0.05).

Most pre-stagnation samples in winter were clustered in the winter cluster, and *Pseudomonas* spp. were dominant in these samples, with a relative abundance of 37–59%. After stagnation, *Pseudomonas* spp. were still dominant at several faucets (F1, F3, F4, and F5). F7 and F8 were grouped in post-stagnation cluster 2 after stagnation in winter, and the relative abundance of *Pseudomonas* spp. was higher (16–27%) than that of the other samples.

### Occurrence of opportunistic pathogens

**Fig. 4.**
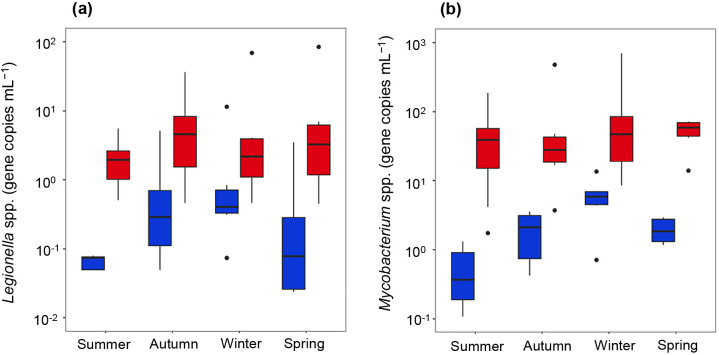
Gene copy numbers of (a) *Legionella* spp. and (b) *Mycobacterium* spp. in (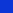) pre- and (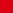) post-stagnation samples (first 1 L) collected in four seasons.

The abundance of opportunistic pathogens was quantified by real-time PCR. Clinically important opportunistic pathogens, including *M. avium*, *L. pneumophila*, *P. aeruginosa*, and *Acanthamoeba* spp., were below LOQ in all faucets before and after stagnation (LOQ of pre- and post-stagnation samples=5.0×10^–2^ and 5.0×10^–1^ gene copies mL^–1^, respectively). On the other hand, *Legionella* spp. and *Mycobacterium* spp. significantly increased after stagnation in all seasons (Fig. 4). qPCR revealed that *Legionella* spp. were detected in 62% (16/26) of samples collected from several faucets before stagnation. After stagnation, the detection frequency increased to 81% (21/26), and their abundance significantly increased from 9.6±5.0×10^–1^ to 9.7±4.1×10^0^ gene copies mL^–1^ (paired Student’s *t*-test, *P*=8.281×10^–13^). *Mycobacterium* spp. were positive for all samples before and after stagnation (52/52), and their abundance significantly increased from 2.5±0.6×10^0^ to 8.8±3.0×10 gene copies mL^–1^ (paired Student’s *t*-test; *P*=8.658×10^–13^). The abundance of *Legionella* spp. and *Mycobacterium* spp. post-stagnation was lower in summer; however, these differences were not significant (ANOVA; *P*=0.844 and 0.720, respectively).

**Fig. 5.**
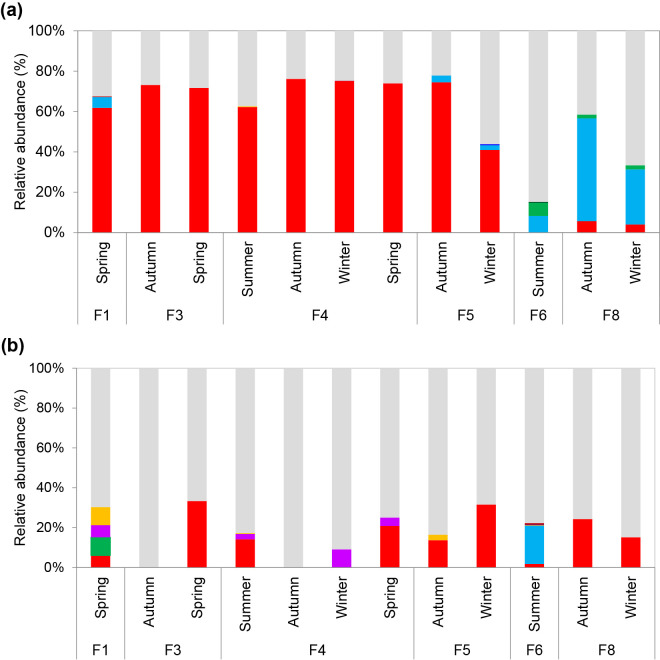
(a) Relative compositions of sequences related to *Legionella* spp. in post-stagnation samples (first 1 L), as analyzed using nanopore sequencing. (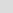) Unassigned *Legionella*, (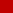) *L. geestiana*, (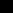) *L. micdadei*, (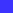) *L. waltersii*, (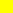) *L. tunisiensis*, (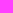) *L. massiliensis*, (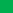) *L. maceachernii*, (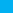) *L. drozanskii*, and (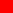) *L. feeleii.* (b) Relative compositions of sequences related to *Mycobacterium* spp. in post-stagnation samples (first 1 L), as analyzed using nanopore sequencing. (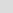) Unassigned *Mycobacterium*, (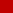) *M. riyadhense*, (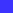) *M. haemophilum*, (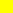) *M. gordonae*, (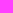) *M. gadium*, (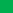) *M. lentiflavum*, (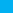) *M. montefiorense*, and (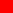) *M. paragordonae.*

Post-stagnation samples with a high abundance of *Legionella* spp. and *Mycobacterium* spp. were subjected to nanopore sequencing targeting full-length 16S rRNA genes in order to obtain detailed phylogenetic information. On average, 60.7% of *Legionella*-related sequences were assigned to species in *Legionella* spp. (Fig. 5). Sequences affiliated with pathogenic *L. feeleii* were the most frequently detected species among the *Legionella* groups, with an average relative abundance of 51.6% of *Legionella*-related sequences. However, the relative abundance of *L. feeleii* at F6 (summer and autumn) and F8 (winter) was less than that of other detected species, such as *L. drozanskii*. Other *Legionella* sequences, which were closely affiliated with pathogenic *L. maceachernii* and *L. micdadei*, were also detected with an average relative abundance of 0.9 and 0.02% of all *Legionella*-related sequences, respectively. On the other hand, only 18.7% of *Mycobacterium*-related sequences were assigned to species in *Mycobacterium* spp. (Fig. 5). Sequences affiliated with pathogenic *M. paragordonae* were the most abundant species, with an average relative abundance of 13.4% of *Mycobacterium-*related sequences. Other *Mycobacterium*-related sequences affiliated with pathogenic *M. gordonae* and *M. haemophilum* were also detected, with an average relative abundance of 1.0 and 0.02% of *Mycobacterium-*related sequences, respectively.

## Discussion

A decrease in free chlorine and increase in TCC after stagnation were observed in all sampling events, which indicated that the regrowth phenomenon at a faucet was common in different faucets in a building. Previous studies demonstrated that the decay of free chlorine after stagnation triggered microbial regrowth (Ling *et al.*, 2018; Dias *et al.*, 2019; Zhang *et al.*, 2021b). The decay of free chlorine after overnight stagnation resulted in an increase in TCC up to 1.8×10^5^‍ ‍cells‍ ‍mL^–1^ (Zhang *et al.*, 2021b). The effect of stagnation on microbial regrowth was also reported in a non-chlorinated system in which increases in intact cell counts (1.1×10^5^‍ ‍cells‍ ‍mL^–1^) and HPC (8.7×10^2^ CFU mL^–1^) were observed after overnight stagnation (Lautenschlager *et al.*, 2010). The higher temperatures of the samples after stagnation than those before stagnation may have accelerated microbial regrowth and the decay of free chlorine. A previous study reported that the chlorine decay rate was higher at 19.4°C than at 15.7°C (García-Ávila *et al.*, 2020). The increase in TCC after stagnation may be attributed to the detachment of bacteria from biofilms on the pipe wall (Manuel *et al.*, 2010). Small-diameter pipes at the distal ends of a building were shown to enhance the dispersal of bacteria from pipe-surface biofilms (Ling *et al.*, 2018). In addition, biodegradable organic matter contained in drinking water or migrating from the pipe material (*e.g.*, polyethylene-coated stainless-steel) has been suggested to affect the regrowth of bacteria in drinking water (Rahmatika *et al.*, 2020).

Seasonal variations in microbial regrowth were assessed in eight faucets (F1–F8) from which samples were collected in four different seasons. TCC levels after stagnation were not significantly different between seasons. Higher TCC in the first 100‍ ‍mL were consistently found in all samples, which indicated that the last meter of premise plumbing was vulnerable to microbial regrowth.

Seasonal differences in microbial richness and diversity were observed in the present study, in which richness (observed OTUs) and the Shannon diversity index were significantly lower in winter. Previous studies also observed lower bacterial richness in winter and variations in the microbial community structure over time (Pinto *et al.*, 2014; Ling *et al.*, 2016). These findings may be attributed to seasonal variations in the microbial community in the source water and growth conditions in premise plumbing.

Pre-stagnation samples in summer, autumn, and spring were dominated by *Phreatobacter* spp., which was consistent with previous findings showing the dominance of *Phreatobacter* spp. in a drinking water system (Perrin *et al.*, 2019a; Van Assche *et al.*, 2019). The lower abundance of *Phreatobacter* spp. in winter than in other seasons was consistent with the finding that *Phreatobacter* spp. were more abundant in the warm period (water temperature >15°C) than in the cold period (water temperature <15°C) in the drinking water distribution system in Paris (Perrin *et al.*, 2019a).

A shift in dominant bacteria from *Phreatobacter* spp. to *Sphingomonas* spp. after stagnation was observed in F1, F2, F4, F5, and F6. A previous study reported that *Sphingomonas* spp. increased from 0 to 10% following the overnight stagnation of tap water (Zhang *et al.*, 2021b). *Sphingomonas* spp. are resistant to chlorine and may develop biofilms along the pipe wall (Sun *et al.*, 2013; Douterelo *et al.*, 2014; Liu *et al.*, 2014; Chao *et al.*, 2015). *Sphingomonas* spp. in biofilms may grow rapidly once the water quality condition changes due to stagnation. *Sphingomonas* have been shown to secrete exopolysaccharides, which are the major components of biofilms (Johnsen *et al.*, 2000; Gulati and Ghosh, 2017). Although biofilms were not directly analyzed in the present study, they may play an important role in microbial regrowth in premise plumbing (Liu *et al.*, 2018; Peng *et al.*, 2020). F3, F7, and F8 after stagnation were dominated by *Comamonadaceae*, *Dechloromonas* spp., *Cyanobacteria*, and *Porphyrobacter* spp., which are commonly found in drinking water distribution systems (Williams *et al.*, 2005; Revetta *et al.*, 2011; Lautenschlager *et al.*, 2013). These results suggest that microbial communities after stagnation are site-specific and dependent on environmental factors, such as water usage frequency. On the other hand, pre- and post-stagnation samples in winter were dominated by *Pseudomonas* spp. The abundance of *Pseudomonas* spp. was higher in the cold period than in the warm period in a drinking water distribution system in Paris (Perrin *et al.*, 2019a). Therefore, *Pseudomonas* spp. have the potential to regrow under low-temperature conditions.

Clinically important opportunistic pathogens, including *M. avium*, *L. pneumophila*, *P. aeruginosa*, and *Acanthamoeba* spp., were below LOQ in all faucets before and after stagnation. Although previous studies reported the presence of these opportunistic pathogens in premise plumbing, the present study revealed that clinically important opportunistic pathogens were below LOQ, regardless of repetitive stagnation events that occurred in the premise plumbing of the building (Wang *et al.*, 2012; Dilger *et al.*, 2018; Liu *et al.*, 2019; Perrin *et al.*, 2019b; Buse *et al.*, 2020; Huang *et al.*, 2021).

On the other hand, *Legionella* spp. and *Mycobacterium* spp. significantly increased after stagnation in all seasons. The abundance of *Legionella* spp. and *Mycobacterium* spp. post-stagnation was lower in summer; however, these differences were not significant. The abundance of *Legionella* spp. and *Mycobacterium* spp. was lower in the present study than in a previous study on household tap water in northern China, which reported the occurrence of *Legionella* spp. and *Mycobacterium* spp., with an average concentration of 6×10^2^ and 4×10^2^ gene copies mL^–1^, respectively (Liu *et al.*, 2019). Despite the similar range of the free chlorine residual in the present study, different water characteristics and water usage practices may explain differences in the abundance of *Legionella* spp. and *Mycobacterium* spp. in premise plumbing. Previous studies reported the abundance of *Legionella* spp. in a hot water system (Leoni *et al.*, 2005; Dilger *et al.*, 2018). Since high water temperatures up to 60°C are beneficial for their growth (Leoni *et al.*, 2005), future studies on hot water supply lines are warranted.

The abundance of *Legionella* spp. and *Mycobacterium* spp. was significantly higher in the first draw than in post-flushing samples (Wang *et al.*, 2012). A positive correlation between *Legionella* spp. and stagnation times was also previously reported (Ley *et al.*, 2020). These findings suggest that *Legionella* spp. and *Mycobacterium* spp. increased after stagnation, and, thus, flushing prior to water consumption may reduce the exposure risk from these genera containing opportunistic pathogens, particularly during the COVID-19 pandemic where the occupancy of buildings has markedly decreased (Hozalski *et al.*, 2020). The increase observed in the abundance of *Legionella* spp. and *Mycobacterium* spp. after stagnation may be associated with biofilms due to their slow growth rates and tendency to form biofilms (Wang *et al.*, 2013). *Mycobacterium* spp. have a hydrophobic cell wall, which protects them from disinfectants and promotes surface attachment and biofilm formation (Steed and Falkinham, 2006). Another important factor influencing the occurrence of *Mycobacterium* spp. is their resistance to the disinfectant. In addition, *Legionella* spp. and *Mycobacterium* spp. both survive and live inside free-living amoeba, such as *Acanthamoeba* spp., which protects them from stress and promotes their growth (Declerck *et al.*, 2005; Lau and Ashbolt, 2009; Liu *et al.*, 2019). *Acanthamoeba* spp. were not detected in the present study, which may have been due to their presence in biofilms. Other hosts, such as *Vermamoeba* spp., need to be investigated in future studies.

Species other than *L. pneumophila* and *M. avium* are also human pathogens (*e.g.*, *L. feeleii*, *L. micdadei*, *M. kansasii*, and *M. abscessus*) (Thomas *et al.*, 1992; Mogami *et al.*, 2016; Sfeir *et al.*, 2018; Wang *et al.*, 2019). Therefore, nanopore sequencing targeting full-length 16S rRNA genes was applied to obtain detailed phylogenetic information. In the present study, sequences affiliated with pathogenic *L. feeleii* were the most frequently detected among *Legionella*-related sequences. The difference observed in the relative abundance of *L. feeleii* among faucets indicated that the composition of *Legionella* spp. was specific at different faucets, even in the same building. *L. feeleii* causes Legionnaires’ disease or Pontiac fever (Wang *et al.*, 2019). It has been detected in several water systems in various countries, such as a hot spring in Taiwan, hot tap water in Germany, and river water being used as a source of drinking water in Japan (Hsu *et al.*, 2006; Dilger *et al.*, 2018; Edagawa *et al.*, 2019). Other *Legionella* sequences affiliated with pathogenic *L. maceachernii* and *L. micdadei* were also detected. *L. maceachernii*, which was initially isolated from a portable water system, caused pneumonia in an immunocompromised patient, while *L. micdadei* caused Legionnaires’ disease or Pontiac fever (Thomas *et al.*, 1992; Wang *et al.*, 2019).

The percentage of *Mycobacterium*-related sequences assigned to species was low in the present study. This may be because 16S rRNA genes among different *Mycobacterium* species were very similar (van der Wielen *et al.*, 2013). Therefore, the more detailed characterization of *Mycobacterium* in premise plumbing is needed. *Mycobacterium* communities may be characterized by analyzing *hsp65* and *rpoB* genes, which demonstrate better resolution in discriminating between species and sub-species (van der Wielen *et al.*, 2013; Haig *et al.*, 2018). Several *Mycobacterium* sequences affiliated with pathogenic *M. paragordonae*, *M. gordonae*, and *M. haemophilum* were detected. *M. paragordonae* was reported to cause peritonitis in a peritoneal dialysis patient, while *M. gordonae* and *M. haemophilum* caused pulmonary and skin infections in elderly immunocompetent patients, respectively (September *et al.*, 2004; Lindeboom *et al.*, 2011; Cheung *et al.*, 2017). Even though clinically important *L. pneumophila* and *M. avium* were not detected in the present study, the potential risks of other sequences that are closely related to pathogenic *Legionella* and *Mycobacterium* need to be intensively monitored.

## Conclusions

Microbial water quality was monitored for one year before and after stagnation in different faucets of the premise plumbing in a building. The main conclusions of the present study are as follows: (1) The depletion of free chlorine was observed in all faucets after stagnation, which resulted in a significant increase in TCC from 10^3^ to 10^4^–10^5^‍ ‍cells‍ ‍mL^–1^. (2) Distinct changes in community structures were observed before and after stagnation because the dominant species changed from *Phreatobacter* spp. to *Sphingomonas* spp., *Comamonadaceae*, *Dechloromonas* spp., *Cyanobacteria*, and *Porphyrobacter* spp. (3) Seasonal variations appeared to play a role in shaping the composition of the microbial community in drinking water, particularly in winter when *Pseudomonas* spp. were dominant. (4) Although clinically important opportunistic pathogens were not detected, several sequences, which were closely related to pathogenic *Legionella* spp. and *Mycobacterium* spp., were identified. Further guidelines regarding water usage practices in premise plumbing are essential for maintaining the good microbial water quality of drinking water prior to consumption.

## Citation

Rahmatika, I., Kurisu, F., Furumai, H., and Kasuga, I. (2022) Dynamics of the Microbial Community and Opportunistic Pathogens after Water Stagnation in the Premise Plumbing of a Building. *Microbes Environ ***37**: ME21065.

https://doi.org/10.1264/jsme2.ME21065

## Supplementary Material

Supplementary Material

## References

[B1] Anaissie, E.J., Penzak, S.R., and Dignani, M.C. (2002) The hospital water supply as a source of nosocomial infections: A plea for action. Arch Intern Med 162: 1483–1492. 12090885 10.1001/archinte.162.13.1483

[B2] APHA. (1998) *Standard Methods for the Examination of Water and Wastewater*. Washington, DC: American Public Health Association.

[B3] Bédard, E., Laferriére, C., Déziel, E., and Prévost, M. (2018) Impact of stagnation and sampling volume on water microbial quality monitoring in large buildings. PLoS One 13: e0199429. 29928013 10.1371/journal.pone.0199429 PMC6013212

[B4] Bolyen, E., Rideout, J.R., Dillon, M.R., Bokulich, N.A., Abnet, C.C., Al-Ghalith, G.A., et al. (2019) Reproducible, interactive, scalable and extensible microbiome data science using QIIME 2. Nat Biotechnol 37: 852–857. 31341288 10.1038/s41587-019-0209-9 PMC7015180

[B5] Buse, H.Y., Morris, B.J., Gomez-Alvarez, V., Szabo, J.G., and Hall, J.S. (2020) Legionella diversity and spatiotemporal variation in the occurrence of opportunistic pathogens within a large building water system. Pathogens 9: 567. 10.3390/pathogens9070567 PMC7400177 32668779

[B6] Caporaso, J.G., Lauber, C.L., Walters, W.A., Berg–Lyons, D., Lozupone, C.A., Turnbaugh, P.J., et al. (2011) Global patterns of 16S rRNA diversity at a depth of millions of sequences per sample. Proc Natl Acad Sci U S A 108 Suppl 1: 4516–4522. 20534432 10.1073/pnas.1000080107 PMC3063599

[B7] Chao, Y., Mao, Y., Wang, Z., and Zhang, T. (2015) Diversity and functions of bacterial community in drinking water biofilms revealed by high–throughput sequencing. Sci Rep 5: 10044. 26067561 10.1038/srep10044 PMC4464384

[B8] Cheung, C.Y., Cheng, N.H.Y., Ting, W.M., and Chak, W.L. (2017) Mycobacterium paragordonae: a rare cause of peritonitis in a peritoneal dialysis patient. Clin Nephrol 88: 371–372. 29057736 10.5414/CN109272

[B9] Declerck, P., Behets, J., Delaedt, Y., Margineanu, A., Lammertyn, E., and Ollevier, F. (2005) Impact of non–Legionella bacteria on the uptake and intracellular replication of Legionella pneumophila in Acanthamoeba castellanii and Naegleria lovaniensis. Microb Ecol 50: 536–549. 16341636 10.1007/s00248-005-0258-0

[B10] Dias, V.C.F., Durand, A.A., Constant, P., Prévost, M., and Bédard, E. (2019) Identification of factors affecting bacterial abundance and community structures in a full–scale chlorinated drinking water distribution system. Water (Basel, Switzerland) 11: 627.

[B11] Dilger, T., Melzl, H., and Gessner, A. (2018) Legionella contamination in warm water systems: A species-level survey. Int J Hyg Environ Health 221: 199–210. 29108681 10.1016/j.ijheh.2017.10.011

[B12] Douterelo, I., Sharpe, R., and Boxall, J. (2014) Bacterial community dynamics during the early stages of biofilm formation in a chlorinated experimental drinking water distribution system: implications for drinking water discolouration. J Appl Microbiol 117: 286–301. 24712449 10.1111/jam.12516 PMC4282425

[B13] Edagawa, A., Kimura, A., and Miyamoto, H. (2019) Investigations on contamination of environmental water samples by *Legionella *using real–time quantitative PCR combined with amoebic co–culturing. Biocontrol Sci 24: 213–220. 31875613 10.4265/bio.24.213

[B14] Falkinham, J.O., 3rd, Iseman, M.D., de Haas, P., and van Soolingen, D. (2008) Mycobacterium avium in a shower linked to pulmonary disease. J Water Health 6: 209–213. 18209283 10.2166/wh.2008.032

[B15] Feazel, L.M., Baumgartner, L.K., Peterson, K.L., Frank, D.N., Harris, J.K., and Pace, N.R. (2009) Opportunistic pathogens enriched in showerhead biofilms. Proc Natl Acad Sci U S A 106: 16393–16399. 19805310 10.1073/pnas.0908446106 PMC2752528

[B16] García-Ávila, F., Sánchez-Alvarracín, C., Cadme-Galabay, M., Conchado-Martínez, J., García-Mera, G., and Zhindón-Arévalo, C. (2020) Relationship between chlorine decay and temperature in the drinking water. MethodsX 7: 101002. 32775225 10.1016/j.mex.2020.101002 PMC7397693

[B17] Gensberger, E.T., Sessitsch, A., and Kostic, T. (2013) Propidium monoazide–quantitative polymerase chain reaction for viable Escherichia coli and Pseudomonas aeruginosa detection from abundant background microflora. Anal Biochem 441: 69–72. 23756735 10.1016/j.ab.2013.05.033

[B18] Gulati, P., and Ghosh, M. (2017) Biofilm forming ability of Sphingomonas paucimobilis isolated from community drinking water systems on plumbing materials used in water distribution. J Water Health 15: 942–954. 29215358 10.2166/wh.2017.294

[B19] Haig, S.J., Kotlarz, N., LiPuma, J.J., and Raskin, L. (2018) A high–throughput approach for identification of nontuberculous mycobacteria in drinking water reveals relationship between water age and *Mycobacterium avium*. mBio 9: e02354–02317. 29440575 10.1128/mBio.02354-17 PMC5821076

[B20] Herpers, B.L., de Jongh, B.M., van der Zwaluw, K., and van Hannen, E.J. (2003) Real–time PCR assay targets the 23S–5S spacer for direct detection and differentiation of Legionella spp. and Legionella pneumophila. J Clin Microbiol 41: 4815–4816. 14532229 10.1128/JCM.41.10.4815-4816.2003 PMC254366

[B21] Hoekstra, E.J., and Tsvetanova, Z.G. (2010) The effect of the surface–to–volume contact ratio on the biomass production potential of the pipe products in contact with drinking water. Water Sci Technol Water Supply 10: 105–112.

[B22] Hozalski, R.M., LaPara, T.M., Zhao, X., Kim, T., Waak, M.B., Burch, T., and McCarty, M. (2020) Flushing of stagnant premise water systems after the COVID–19 shutdown can reduce infection risk by Legionella and Mycobacterium spp. Environ Sci Technol 54: 15914–15924. 33232602 10.1021/acs.est.0c06357

[B23] Hsu, B.M., Chen, C.H., Wan, M.T., and Cheng, H.W. (2006) Legionella prevalence in hot spring recreation areas of Taiwan. Water Res 40: 3267–3273. 16928391 10.1016/j.watres.2006.07.007

[B24] Huang, J., Chen, S., Ma, X., Yu, P., Zuo, P., Shi, B., et al. (2021) Opportunistic pathogens and their health risk in four full–scale drinking water treatment and distribution systems. Ecol Eng 160: 106134.

[B25] Johnsen, A.R., Hausner, M., Schnell, A., and Wuertz, S. (2000) Evaluation of fluorescently labeled lectins for noninvasive localization of extracellular polymeric substances in Sphingomonas biofilms. Appl Environ Microbiol 66: 3487–3491. 10919811 10.1128/aem.66.8.3487-3491.2000 PMC92175

[B26] Lau, H.Y., and Ashbolt, N.J. (2009) The role of biofilms and protozoa in Legionella pathogenesis: implications for drinking water. J Appl Microbiol 107: 368–378. 19302312 10.1111/j.1365-2672.2009.04208.x

[B27] Lautenschlager, K., Boon, N., Wang, Y., Egli, T., and Hammes, F. (2010) Overnight stagnation of drinking water in household taps induces microbial growth and changes in community composition. Water Res 44: 4868–4877. 20696451 10.1016/j.watres.2010.07.032

[B28] Lautenschlager, K., Hwang, C., Liu, W.T., Boon, N., Koster, O., Vrouwenvelder, H., et al. (2013) A microbiology–based multi–parametric approach towards assessing biological stability in drinking water distribution networks. Water Res 47: 3015–3025. 23557697 10.1016/j.watres.2013.03.002

[B29] Leoni, E., De Luca, G., Legnani, P.P., Sacchetti, R., Stampi, S., and Zanetti, F. (2005) Legionella waterline colonization: detection of Legionella species in domestic, hotel and hospital hot water systems. J Appl Microbiol 98: 373–379. 15659192 10.1111/j.1365-2672.2004.02458.x

[B30] Ley, C.J., Proctor, C.R., Singh, G., Ra, K., Noh, Y., Odimayomi, T., et al. (2020) Drinking water microbiology in a water–efficient building: stagnation, seasonality, and physicochemical effects on opportunistic pathogen and total bacteria proliferation. Environ Sci: Water Res Technol 6: 2902–2913.

[B31] Lindeboom, J.A., Bruijnesteijn van Coppenraet, L.E., van Soolingen, D., Prins, J.M., and Kuijper, E.J. (2011) Clinical manifestations, diagnosis, and treatment of Mycobacterium haemophilum infections. Clin Microbiol Rev 24: 701–717. 21976605 10.1128/CMR.00020-11 PMC3194825

[B32] Ling, F., Hwang, C., LeChevallier, M.W., Andersen, G.L., and Liu, W.T. (2016) Core–satellite populations and seasonality of water meter biofilms in a metropolitan drinking water distribution system. ISME J 10: 582–595. 26251872 10.1038/ismej.2015.136 PMC4817684

[B33] Ling, F., Whitaker, R., LeChevallier, M.W., and Liu, W.T. (2018) Drinking water microbiome assembly induced by water stagnation. ISME J 12: 1520–1531. 29588495 10.1038/s41396-018-0101-5 PMC5955952

[B34] Liu, G., Bakker, G.L., Li, S., Vreeburg, J.H., Verberk, J.Q., Medema, G.J., et al. (2014) Pyrosequencing reveals bacterial communities in unchlorinated drinking water distribution system: an integral study of bulk water, suspended solids, loose deposits, and pipe wall biofilm. Environ Sci Technol 48: 5467–5476. 24766451 10.1021/es5009467

[B35] Liu, G., Zhang, Y., van der Mark, E., Magic-Knezev, A., Pinto, A., van den Bogert, B., et al. (2018) Assessing the origin of bacteria in tap water and distribution system in an unchlorinated drinking water system by SourceTracker using microbial community fingerprints. Water Res 138: 86–96. 29573632 10.1016/j.watres.2018.03.043

[B36] Liu, L., Xing, X., Hu, C., and Wang, H. (2019) One-year survey of opportunistic premise plumbing pathogens and free-living amoebae in the tap-water of one northern city of China. J Environ Sci 77: 20–31. 10.1016/j.jes.2018.04.020 30573084

[B37] Liu, M., Hata, A., Katayama, H., and Kasuga, I. (2020) Consecutive ultrafiltration and silica adsorption for recovery of extracellular antibiotic resistance genes from an urban river. Environ Pollut 260: 114062. 32041028 10.1016/j.envpol.2020.114062

[B38] Manuel, C.M., Nunes, O.C., and Melo, L.F. (2010) Unsteady state flow and stagnation in distribution systems affect the biological stability of drinking water. Biofouling 26: 129–139. 19859848 10.1080/08927010903383448

[B39] Mogami, R., Goldenberg, T., de Marca, P.G., Mello, F.C., and Lopes, A.J. (2016) Pulmonary infection caused by Mycobacterium kansasii: findings on computed tomography of the chest. Radiol Bras 49: 209–213. 27777472 10.1590/0100-3984.2015.0078 PMC5073385

[B40] Peng, H., Zhang, Y., Wang, R., Liu, J., and Liu, W.T. (2020) Assessing the contribution of biofilm to bacterial growth during stagnation in shower hoses. Water Sci Technol Water Supply 20: 2564–2576.

[B41] Perrin, Y., Bouchon, D., Delafont, V., Moulin, L., and Hechard, Y. (2019a) Microbiome of drinking water: A full–scale spatio–temporal study to monitor water quality in the Paris distribution system. Water Res 149: 375–385. 30471533 10.1016/j.watres.2018.11.013

[B42] Perrin, Y., Bouchon, D., Hechard, Y., and Moulin, L. (2019b) Spatio–temporal survey of opportunistic premise plumbing pathogens in the Paris drinking water distribution system. Int J Hyg Environ Health 222: 687–694. 31085113 10.1016/j.ijheh.2019.04.010

[B43] Pinto, A.J., Schroeder, J., Lunn, M., Sloan, W., and Raskin, L. (2014) Spatial–temporal survey and occupancy–abundance modeling to predict bacterial community dynamics in the drinking water microbiome. mBio 5: e01135–14. 24865557 10.1128/mBio.01135-14 PMC4045074

[B44] Prest, E.I., Hammes, F., van Loosdrecht, M.C., and Vrouwenvelder, J.S. (2016) Biological stability of drinking water: Controlling factors, methods, and challenges. Front Microbiol 7: 45. 26870010 10.3389/fmicb.2016.00045 PMC4740787

[B45] Proctor, C.R., and Hammes, F. (2015) Drinking water microbiology–from measurement to management. Curr Opin Biotechnol 33: 87–94. 25578740 10.1016/j.copbio.2014.12.014

[B46] Rahmatika, I., Kasuga, I., Kurisu, F., and Furumai, H. (2020) Impacts of organic matter migrating from pipe materials on microbial regrowth in drinking water. J Water Environ Technol 18: 45–53.

[B47] Revetta, R.P., Matlib, R.S., and Santo Domingo, J.W. (2011) 16S rRNA gene sequence ana­lysis of drinking water using RNA and DNA extracts as targets for clone library development. Curr Microbiol 63: 50–59. 21533782 10.1007/s00284-011-9938-9

[B48] Rhoads, W.J., and Hammes, F. (2021) Growth of Legionella during COVID-19 lockdown stagnation. Environ Sci: Water Res Technol 7: 10–15.

[B49] Riviere, D., Szczebara, F.M., Berjeaud, J.M., Frere, J., and Hechard, Y. (2006) Development of a real–time PCR assay for quantification of Acanthamoeba trophozoites and cysts. J Microbiol Methods 64: 78–83. 15923051 10.1016/j.mimet.2005.04.008

[B50] September, S.M., Brozel, V.S., and Venter, S.N. (2004) Diversity of nontuberculoid Mycobacterium species in biofilms of urban and semiurban drinking water distribution systems. Appl Environ Microbiol 70: 7571–7573. 15574964 10.1128/AEM.70.12.7571-7573.2004 PMC535200

[B51] Sfeir, M., Walsh, M., Rosa, R., Aragon, L., Liu, S.Y., Cleary, T., et al. (2018) Mycobacterium abscessus complex infections: A retrospective cohort study. Open Forum Infect Dis 5: ofy022. 29450214 10.1093/ofid/ofy022 PMC5808791

[B52] Steed, K.A., and Falkinham, J.O., 3rd. (2006) Effect of growth in biofilms on chlorine susceptibility of Mycobacterium avium and Mycobacterium intracellulare. Appl Environ Microbiol 72: 4007–4011. 16751509 10.1128/AEM.02573-05 PMC1489660

[B53] Sun, W., Liu, W., Cui, L., Zhang, M., and Wang, B. (2013) Characterization and identification of a chlorine–resistant bacterium, Sphingomonas TS001, from a model drinking water distribution system. Sci Total Environ 458–460: 169–175. 10.1016/j.scitotenv.2013.04.030 23648446

[B54] Thomas, E., Gupta, N.K., van der Westhuizen, N.G., Chan, E., and Bernard, K. (1992) Fatal Legionella maceachernii pneumonia in Canada. J Clin Microbiol 30: 1578–1579. 1624578 10.1128/jcm.30.6.1578-1579.1992 PMC265333

[B55] Van Assche, A., Crauwels, S., De Brabanter, J., Willems, K.A., and Lievens, B. (2019) Characterization of the bacterial community composition in water of drinking water production and distribution systems in Flanders, Belgium. MicrobiologyOpen 8: e00726. 30318762 10.1002/mbo3.726 PMC6528567

[B56] van der Wielen, P.W., Heijnen, L., and van der Kooij, D. (2013) Pyrosequence ana­lysis of the hsp65 genes of nontuberculous mycobacterium communities in unchlorinated drinking water in the Netherlands. Appl Environ Microbiol 79: 6160–6166. 23913420 10.1128/AEM.01591-13 PMC3811344

[B57] Wang, C., Chuai, X., and Liang, M. (2019) Legionella feeleii: pneumonia or Pontiac fever? Bacterial virulence traits and host immune response. Med Microbiol Immunol 208: 25–32. 30386929 10.1007/s00430-018-0571-0

[B59] Wang, H., Edwards, M., Falkinham, J.O., 3rd, and Pruden, A. (2012) Molecular survey of the occurrence of Legionella spp., Mycobacterium spp., Pseudomonas aeruginosa, and amoeba hosts in two chloraminated drinking water distribution systems. Appl Environ Microbiol 78: 6285–6294. 22752174 10.1128/AEM.01492-12 PMC3416603

[B60] Wang, H., Edwards, M.A., Falkinham, J.O., 3rd, and Pruden, A. (2013) Probiotic approach to pathogen control in premise plumbing systems? A review. Environ Sci Technol 47: 10117–10128. 23962186 10.1021/es402455r

[B61] Wen, G., Kotzsch, S., Vital, M., Egli, T., and Ma, J. (2015) BioMig–A method to evaluate the potential release of compounds from and the formation of biofilms on polymeric materials in contact with drinking water. Environ Sci Technol 49: 11659–11669. 26338053 10.1021/acs.est.5b02539

[B62] Williams, M.M., Santo Domingo, J.W., and Meckes, M.C. (2005) Population diversity in model potable water biofilms receiving chlorine or chloramine residual. Biofouling 21: 279–288. 16522541 10.1080/08927010500452695

[B63] Yoon, S.-H., Ha, S.-M., Kwon, S., Lim, J., Kim, Y., Seo, H., and Chun, J. (2017) Introducing EzBioCloud: a taxonomically united database of 16S rRNA gene sequences and whole-genome assemblies. Int J Syst Evol Microbiol 67: 1613–1617. 28005526 10.1099/ijsem.0.001755 PMC5563544

[B64] Zhang, H., Xu, L., Huang, T., Liu, X., Miao, Y., Liu, K., and Qian, X. (2021a) Indoor heating triggers bacterial ecological links with tap water stagnation during winter: Novel insights into bacterial abundance, community metabolic activity and interactions. Environ Pollut 269: 116094. 33234370 10.1016/j.envpol.2020.116094

[B65] Zhang, H., Xu, L., Huang, T., Yan, M., Liu, K., Miao, Y., et al. (2021b) Combined effects of seasonality and stagnation on tap water quality: Changes in chemical parameters, metabolic activity and co-existence in bacterial community. J Hazard Mater 403: 124018. 33265044 10.1016/j.jhazmat.2020.124018

[B66] Zlatanovic, L., van der Hoek, J.P., and Vreeburg, J.H.G. (2017) An experimental study on the influence of water stagnation and temperature change on water quality in a full-scale domestic drinking water system. Water Res 123: 761–772. 28732329 10.1016/j.watres.2017.07.019

