# Supplementary material for: Dynamics of the Microbial Community and Opportunistic Pathogens after Water Stagnation in the Premise Plumbing of a Building
Source: Microbes Environ. 2022 Mar 24;37(1):ME21065. doi: 10.1264/jsme2.ME21065 (PMC8958293; doi:10.1264/jsme2.ME21065)
Supplement: Supplementary file 1 — Supplementary Material [file 37_21065_s1.pdf]

Supplementary information

Dynamics of the Microbial Community and Opportunistic Pathogens after Water Stagnation in the Premise Plumbing of a Building

Iftita Rahmatika[1], Futoshi Kurisu[2], Hiroaki Furumai[2] & Ikuro Kasuga[1*]

[1]Department of Urban Engineering, Graduate School of Engineering, The University of Tokyo, Tokyo, Japan

[2]Research Center for Water Environment Technology, Graduate School of Engineering, The University of Tokyo, Tokyo, Japan

***Corresponding author: Ikuro Kasuga**

Mailing address:

Department of Urban Engineering

Graduate School of Engineering

The University of Tokyo

7-3-1 Hongo, Bunkyo-ku, Tokyo, Japan

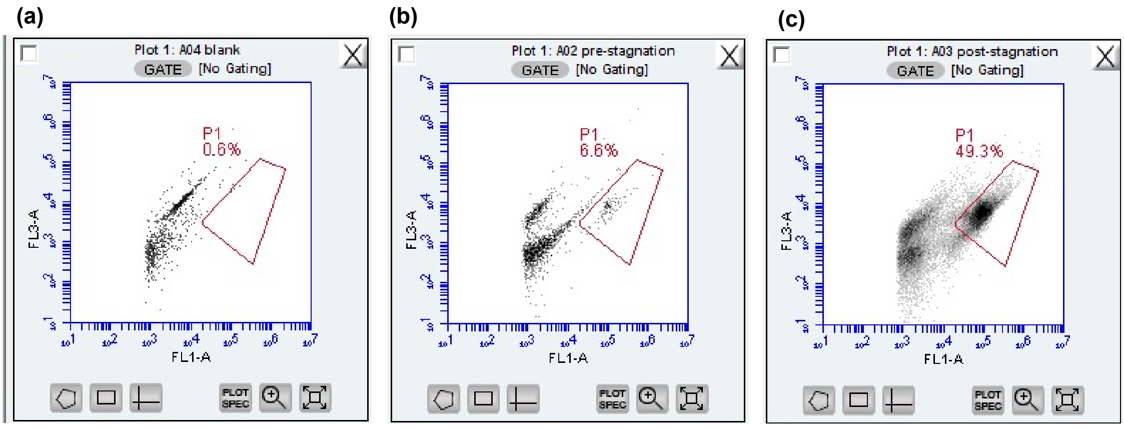

Fig. S1 FCM gating example for (a) blank, (b) pre-, and (c) post-stagnation samples at F1 collected in winter season.

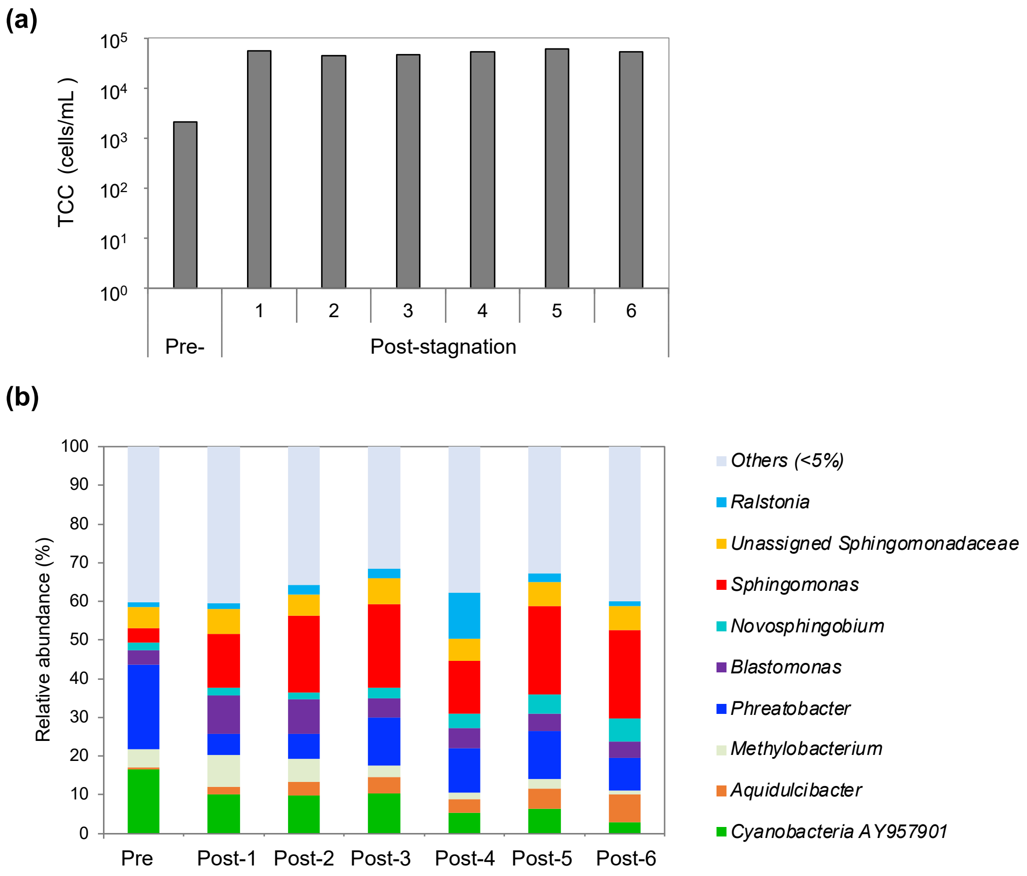

Fig. S2 (a) Total cell counts and (b) microbial community structures at the genus level of the initial pre-stagnation sample and six post-stagnation samples at F1. Pre and Post indicates the initial pre- and post- stagnation samples, respectively. Post 1-6 indicates the respective temporal replicates of post-stagnation samples across 6 consecutive days.

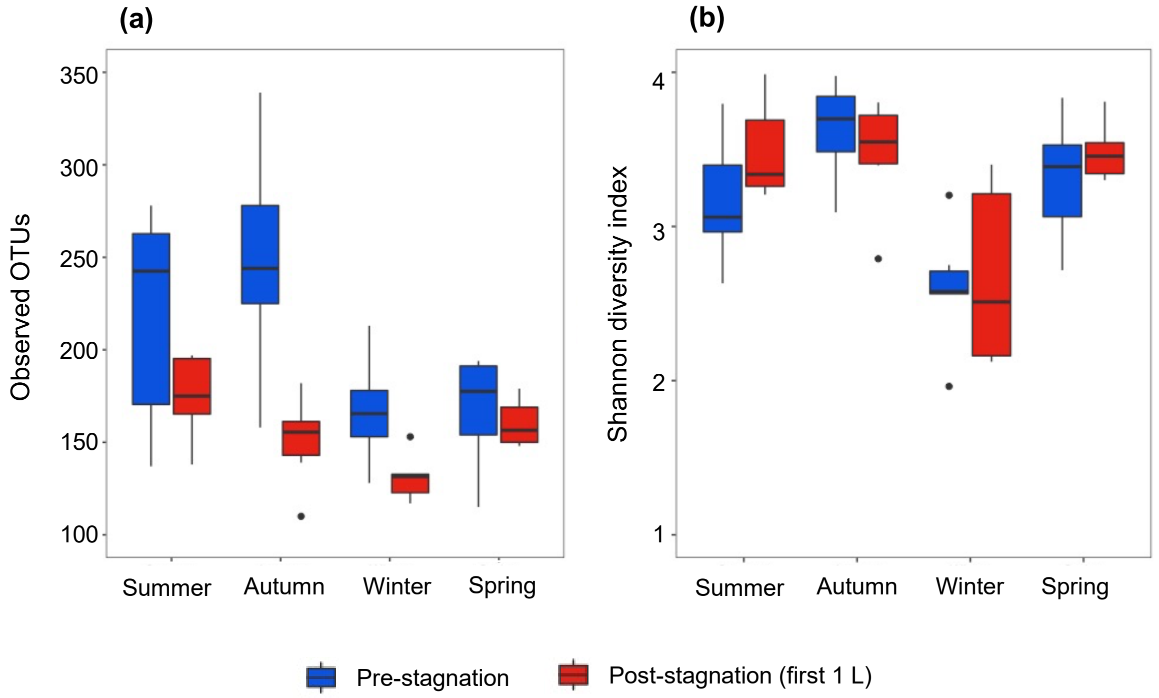

Fig. S3 Seasonal variations in (a) observed OTUs and (b) Shannon diversity index of microbial communities pre- and post-stagnation (first 1 L) samples collected in four seasons.

Table S1 Date of sampling from premise plumbing.

Season	Faucet	Sampling date	
		Pre-stagnation	Post-stagnation
Summer 2018	1	24 May	25 May
	2	22 Jun	23 Jun
	3	21 Jun	23 Jun
	4	26 May	27 May
	5	24 Jun	25 Jun
	6	23 Jun	24 Jun
	7	29 Jun	30 Jun
	8	1 Jul	2 Jul
Autumn 2018	1	15 Nov	16 Nov
	3	6 Dec	7 Dec
	4	15 Nov	16 Nov
	5	27 Nov	28 Nov
	7	23 Nov	24 Nov
	8	25 Nov	26 Nov
Winter 2019	1	21 Jan	22 Jan
	3	23 Jan	24 Jan
	4	24 Jan	25 Jan
	5	25 Jan	26 Jan
	7	26 Jan	27 Jan
	8	26 Jan	27 Jan
Spring 2019	1	19 Apr	20 Apr
	3	13 Apr	14 Apr
	4	14 Apr	15 Apr
	5	16 Apr	17 Apr
	7	16 Apr	17 Apr
	8	20 Apr	21 Apr

Table S2 Real-time PCR primers and probes used in this study.

Target	Primers and probe name	Sequence (5'-3')	PCR thermoprofile	Reference	PCR efficiencies (%)
Legionella spp. (23S-5S rRNA spacer region)	LegF<br>LegR<br>Leg Probe	CTAATTGGCTGATTGTCTTGAC<br>GGCGATGACCTACTTTCG<br>(FAM)-CGAACTCAGAAGTGAAAC-(MGBNFQ)	95°C for 10 min; 45 cycles of 95°C for 15 sec and 60°C for 1 min.	(Herpers et al. 2003).	95.55–99.50
Legionella pneumophila (mip gene)	LPQF<br>LPQR<br>mip Probe	TTCATTTGYTGYTCGGTTAAAGC<br>AWTGGCTAAAGGCATGCAAGAC<br>(FAM)-AGCGCCACTCATAG-(MGBNFQ)	95°C for 10 min; 45 cycles of 95°C for 15 sec and 60°C for 1 min	(Herpers et al. 2003).	95.55–99.50
M. avium (16S rRNA)	MAV77F<br>MAV199R<br>MavPrC	GCCTCTTCGGAGGTACTCG<br>ACCAGAAGACATGCGTCTTG<br>(VIC)-CAATCTGCCCTGCACTTCGGGATAAG-(MGBNFQ)	95°C for 5 min; 43 cycles of 95°C for 20 sec and 60°C for 1 min	(Feazel et al. 2009).	90.15–98.50
Mycobacterium spp. (16S rRNA)	Myco637f<br>Myco841r<br>Myco662p	GGGCGATACGGGCAGAC<br>GAAACCCACACCTAGTACC<br>(FAM)-CAGGGGAGACTGGAATTCCTGGTGTA-(MGBNFQ)	95°C for 5 min; 43 cycles of 95°C for 20 sec and 60°C for 1 min	(Feazel et al. 2009)	90.15–98.50
Pseudomonas aeruginosa (*regA* gene)	Paer-Fb<br>Paer-Rb<br>Paer-PrA	ATCGAGTACCTGAACCGGC<br>TGGTGCAGTTCCTCATTGTC<br>(FAM)-CCAGATGCTTTGCCTCAAC-(MGBNFQ)	95°C for 2 min; 40 cycles of 95°C for 5 sec and 62°C for 30 sec.	(Gensberger et al. 2013).	92.75–99.25
Acanthamoeba (18S rRNA)	TaqAcF1<br>TaqAcR1<br>Probe TaqAcP1	CGACCAGCGATTAGGAGACG<br>CCGACGCCAAGGACGAC<br>(FAM)-TGAATACAAAACACCACCATCGGCGC-(MGBNFQ)	50°C for 2 min; 40 cycles of 95°C for 15 sec and 62°C for 1 min.	(Riviere et al. 2006).	91.45–95.30

Table S3 Detail information of the target genes included in the artificial plasmid used for real-time quantitative PCR standard.

Target genes	Strain	Accession number
Legionella spp. (23S-5S rRNA spacer region)	*Legionella pneumophila* subsp. *pneumophila* LPE509	CP003885
Legionella pneumophila (mip gene)	*Legionella pneumophila*, Philadelphia 1	S42595
Mycobacterium avium (16S rRNA)	*Mycobacterium avium subsp. avium* ATCC 25291	GQ153272
Mycobacterium spp. (16S rRNA)	*Mycobacterium avium subsp. avium* ATCC 25291	GQ153272
Pseudomonas eruginosa (regA gene)	*Pseudomonas aeruginosa* strain PA0	EU341998
Acanthamoeba spp. (18S rRNA)	*Acanthamoeba castellanii* Castellani ATCC 50374	U07413

Table S4 Water characteristic of pre- and post-stagnation (first 100 mL)

Faucets		Water temperature (°C)			Free chlorine (mg/L)		TCC (cells/mL)		HPC (CFU/mL)	
		Pre-	Post-	Changes	Pre-	Post-	Pre-	Post-	Pre-	Post-
F1	Summer	21.2	22.1	0.9	0.35	<0.02	1.2×10^3	6.7×10^4	ND	4.7×10^3
	Autumn	19.3	22.0	2.7	0.29	<0.02	1.1×10^3	6.3×10^4	ND	2.5×10^3
	Winter	12.8	21.1	8.3	0.32	<0.02	9.0×10^2	3.5×10^4	ND	2.3×10^3
	Spring	18.3	20.7	2.4	0.29	<0.02	2.1×10^3	5.5×10^4	ND	1.4×10^3
F2	Summer	22.6	22.3	-0.3	0.31	<0.02	4.0×10^2	3.0×10^4	ND	6.9×10^2
F3	Summer	22.3	22.0	-0.3	0.28	<0.02	5.7×10^3	2.2×10^5	ND	9.5×10^3
	Autumn	18.2	22.8	4.6	0.30	<0.02	2.8×10^3	1.5×10^5	ND	7.6×10^3
	Winter	13.2	20.6	7.4	0.32	<0.02	1.8×10^3	9.1×10^4	ND	2.4×10^3
	Spring	16.7	21.1	4,4	0.30	<0.02	1.0×10^3	1.0×10^5	ND	1.6×10^3
F4	Summer	23.7	24.2	0.5	0.29	<0.02	1.3×10^3	4.4×10^4	ND	1.6×10^3
	Autumn	20.7	22.0	1.3	0.33	<0.02	2.4×10^3	5.7×10^4	ND	9.2×10^2
	Winter	13.0	19.5	6.5	0.32	<0.02	2.9×10^3	4.1×10^4	ND	2.3×10^2
	Spring	17.6	21.0	3.4	0.27	<0.02	1.6×10^3	4.1×10^4	ND	5.3×10^1
F5	Summer	23.1	22.9	-0.2	0.27	<0.02	9.0×10^2	7.4×10^4	ND	6.6×10^2
	Autumn	17.3	20.4	3.1	0.29	<0.02	4.0×10^2	8.8×10^4	ND	6.2×10^2
	Winter	12.9	17.5	4.6	0.31	<0.02	3.5×10^3	7.2×10^4	ND	1.4×10^2
	Spring	16.7	20.1	3.4	0.36	<0.02	9.0×10^2	7.6×10^4	ND	1.5×10^2
F6	Summer	23.1	22.7	-0.4	0.27	<0.02	2.4×10^3	8.8×10^4	ND	1.0×10^3
F7	Summer	27.0	27.6	0.6	0.26	<0.02	1.2×10^3	1.8×10^5	ND	5.0×10^2
	Autumn	19.8	18.0	-1.8	0.27	<0.02	5.4×10^3	1.9×10^5	ND	4.7×10^2
	Winter	14.4	20.8	6.4	0.32	<0.02	4.9×10^3	1.8×10^5	ND	6.3×10^2
	Spring	17.2	20.8	3.6	0.34	<0.02	8.0×10^2	1.4×10^5	ND	1.1×10^3
F8	Summer	27.0	27.6	0.6	0.17	<0.02	7.4×10^3	4.2×10^5	ND	5.0×10^2
	Autumn	19.5	20.4	0.9	0.25	<0.02	1.2×10^4	2.9×10^5	ND	5.2×10^2
	Winter	14.7	20.3	6.6	0.28	<0.02	3.1×10^3	1.8×10^5	ND	1.4×10^2
	Spring	20.2	22.0	1.8	0.23	<0.02	2.0×10^3	2.7×10^5	ND	1.6×10^2

ND= not detected

Table S5 *p* values and 95% confidence intervals of paired t-test analysis for pre- and post-stagnation samples.

Parameter	Number of samples (n)	*p* value	95% CI of absolute difference
Temperature (ºC)	26	3.059×10^{-5}	1.62–3.84
TCC (log_{10} cells/mL)	26	8.598×10^{-21}	1.59–3.84
Free chlorine (mg/L)	26	9.738×10^{-23}	0.256–0.288
OTU richness	25	2.632×10^{-4}	22.96–65.84
Legionella spp. (log_{10} gene copies/mL)	26	8.281×10^{-13}	1.00–1.37
Mycobacterium spp. (log gene copies/mL)	26	8.658×10^{-13}	0.69–1.23

Table S6 F values and *p* values of one-way ANOVA analysis for different seasonal samples (spring, summer, autumn, and winter).

Parameter	Number of samples (n)	F value	*p* value
Free chlorine in post-stagnation (mg/L)	6	1.119	0.365
HPC in post-stagnation (log_{10} CFU/mL)	6	0.741	0.540
TCC in pre-stagnation (log_{10} cells/mL)	6	0.775	0.521
TCC in post-stagnation (log_{10} cells/mL)	6	0.427	0.736
OTU richness in pre-stagnation	5	3.856	0.029
OTU richness in post-stagnation	6	8.385	8.300×10^{-4}
Legionella spp. in post-stagnation (log_{10} gene copies/mL)	6	0.273	0.844
Mycobacterium spp. in post-stagnation (log_{10} gene copies/mL)	6	0.450	0.720

Table S7 *p* value of Tukey's HSD statistical analysis.

Parameter	Comparison	*p* value
OTU richness pre-stagnation	Spring-Autumn	0.071
	Summer-Autumn	0.653
	Winter-Autumn	0.039
	Summer-Spring	0.456
	Winter-Spring	0.989
	Winter-Summer	0.302
OTU richness post-stagnation	Spring-Autumn	0.799
	Summer-Autumn	0.033
	Winter-Autumn	0.245
	Summer-Spring	0.193
	Winter-Spring	0.045
	Winter-Summer	4.334×10^{-4}
Shannon diversity index pre-stagnation	Spring-Autumn	0.959
	Summer-Autumn	0.996
	Winter-Autumn	0.020
	Summer-Spring	0.993
	Winter-Spring	0.053
	Winter-Summer	0.031
Shannon diversity index pre-stagnation	Spring-Autumn	1.000
	Summer-Autumn	0.012
	Winter-Autumn	0.767
	Summer-Spring	0.012
	Winter-Spring	0.758
	Winter-Summer	0.001

Table S8 Effect of seasonal variations in the microbial communities of pre- and post-stagnation samples as observed by ANOSIM.

	Pre-stagnation		Post-stagnation water	
	Global R	*p* value	Global R	*p* value
Seasonal variation	0.6951	0.001	0.2661	0.005
Pairwise test				
Summer vs autumn	0.5263	0.002	0.0145	0.343
Summer vs winter	1.0000	0.003	0.6066	0.003
Summer vs spring	0.3023	0.013	0.1056	0.147
Autumn vs winter	0.7707	0.002	0.3981	0.012
Winter vs spring	1.0000	0.003	0.5537	0.003
Autumn vs spring	0.2373	0.032	0.1204	0.859

References

Feazel, L.M., Baumgartner, L.K., Peterson, K.L., Frank, D.N., Harris, J.K. and Pace, N.R. (2009) Opportunistic pathogens enriched in showerhead biofilms. *Proc Natl Acad Sci U S A* **106**(38): 16393–16399.

Gensberger, E.T., Sessitsch, A. and Kostic, T. (2013) Propidium monoazide–quantitative polymerase chain reaction for viable Escherichia coli and Pseudomonas aeruginosa detection from abundant background microflora. *Anal Biochem* **441**(1): 69–72.

Herpers, B.L., de Jongh, B.M., van der Zwaluw, K. and van Hannen, E.J. (2003) Real–time PCR assay targets the 23S–5S spacer for direct detection and differentiation of Legionella spp. and Legionella pneumophila. *J Clin Microbiol* **41**(10): 4815–4816.

Riviere, D., Szczebara, F.M., Berjeaud, J.M., Frere, J. and Hechard, Y. (2006) Development of a real–time PCR assay for quantification of Acanthamoeba trophozoites and cysts. *J Microbiol Methods* **64**(1): 78–83.
